# CHILDHOOD ROOTS OF FRAILTY: MACHINE LEARNING INSIGHTS INTO HEALTH INEQUALITY IN LATER LIFE

**DOI:** 10.1093/geroni/igae098.0598

**Published:** 2024-12-31

**Authors:** Shutong Huo, Thomas Gill, Xi Chen, Derek Feng

**Affiliations:** University of California Irvine, Irvine, California, United States; Yale University, New Haven, Connecticut, United States; Yale University, New Haven, Connecticut, United States; Yale University, New Haven, Connecticut, United States

## Abstract

This study investigates the impact of childhood circumstances on health inequality in later life, with a particular emphasis on frailty among older adults in the United States, highlighting the significance of early life historical and social factors. We employed data from the Health and Retirement Study (HRS), incorporating the 2012, 2014, 2016, and 2018 waves along with the 2015 Life History Mail Survey (LHMS). Using innovative conditional inference trees and forests, we evaluated 43 distinct childhood factors and their contribution to the Inequality of Opportunity (IOP) in health outcomes. The circumstances in both countries can be divided into seven domains: 1) war or economic crisis at birth; 2) regional and urban/rural status at birth; 3) family SES in childhood; 4) parental health status and health behaviors in childhood; 5) health and nutritional status in childhood; 6) relationship with parents in childhood; 7) friendship in childhood. We found that key early-life predictors identified include experiencing the Great Depression, adverse childhood events, socioeconomic status, and access to educational resources, all of which play critical roles in determining frailty in older adults. The machine learning models, particularly conditional inference forests, significantly outperform traditional analytical methods in predicting health inequality, with the best out-of-sample performance. The findings demonstrate the importance of early-life circumstances in shaping later health outcomes and stress the early-life interventions for health equity in aging societies.

